# Her Heart's Mistake

**Published:** 1889-07-20

**Authors:** E. D. Cumming

**Affiliations:** Author of "An Ordeal by Silence"


### Her Heart's Mistake.

By E. D. Cumming, Author of "An Ordeal by Silence."

*(Continued from page 237.)*

### Chapter XIV.—Some Correspondence.

Kate's first feeling, as the recollection of that unwritten letter flashed across her mind, was one of simple vexation, as she could not now get it ready to post at the usual hour next morning. But as she went into her room and prepared to begin, intending to do her best to make up for lost time, her conscience smote her for having thus allowed a stranger to absorb her so completely, that she had not only left this sacred duty undone, but had forgotten it altogether. She did not attempt to justify her neglect to herself, but set to work to atone by writing in a strain of more than usual tenderness ; she was in the mood for it, for her conversation with Warren had stirred her mind to its greatest depths, and her pen flew on untiringly until she could look with satisfaction upon three well-filled sheets of note paper. She forgave herself the lapse of memory which was keeping her up so late, and, laying down her pen, leaned back in her chair to read over the letter before she closed it. No harm was done, after all ; she could send a servant off with it the first thing in the morning and save the post. But as she perused it, her brow contracted with a curious expression of surprise, doubt, or annoyance, which grew more marked as she read on.

"Mr. Warren says, Mr. Warren thinks, Mr. Warren believes, I asked Mr. Warren"— "Why, it's Warren, Warren, in every line," she murmured half aloud, as she put down the letter and stared at it. "What on earth have I been dreaming about? George won't know what to think if I send him such a thing as that. I've quoted Mr. Warren, and referred to him in nearly every sentence."

She took the sheets in both hands and slowly tore them across and across, looking graver and more vexed with herself as she did so. She threw the fragments into the waste paper basket and began again, though it was past midnight and she was tired. She wrote more carefully, and gradually grew engrossed in her work as she went on ; when she had reached the end of the first page she stopped and read over what she had written. That was better ; Mr. Warren's name only appeared once, in the most incidental way, and she resumed her pen with more confidence. But the next page, written with less watchful care, showed Mr. Warren in a matter-of-fact light as though he were a feature of her life, almost as though he had become a member of the household. Kate threw aside her pen and blushed with annoyance. How was it that she could not keep him out of her letter? George would not like to think that another man claimed so large a share of her interest as this barrister appeared to be doing. It would never do to send an epistle which might give him reason to suppose that anyone in the world was anything more than an ordinary acquaintance. And yet, if she rewrote that second sheet, studiously avoiding all reference to Mr. Warren, she would feel that she was purposely keeping something from her lover, when there was no shadow of a reason for doing so. Of course, it was perfectly easy to explain it to him ; how stupid of her not to have thought of it before. Papa liked Mr. Warren, and asked him in often ; so frequently that she saw him nearly every evening. Her interest in him was on her father's account ; and she added a few lines in which she enlarged upon her gratitude for the happy chance which had sent papa a pleasant companion at the time he most felt the need of one. Kate was not entirely satisfied with the letter when it was finished, but she would not write it again : George must know that Mr. Warren could be nothing to her, and if she couldn't mention her friends without annoying him, the sooner she gave up writing to him altogether the better. Then she reproached herself for thinking that George would be so foolish as to take exception to her forming new acquaintances whilst he was away ; it was unfair to him, and she paid him a very poor compliment in supposing for a moment that he would even notice her references to Mr. Warren. But in spite of her self-assurances that she had written nothing to which she need give a moment's uneasy thought, she retired to her room with some tiny doubts still lurking in a corner of her mind.

She received a letter from Simla one afternoon, a day or two later, and went into her own room to read it, with her heart beating a little more rapidly than was it wont. This was the answer to the long one she had written the other night after Mr. Warren went away, and she was not quite happy about it still. However, George had replied as promptly as usual, and she opened the envelope without hesitation. She felt the blood rush to her face as she read the first few lines ; they were written in a querulous, almost sarcastic vein which fairly astonished her.

"I am glad to learn that the Doctor has found a congenial companion," Bairdsley wrote. "You seem to think very highly of him, too, judging by the tone of your letter. Of course, I know you must have friends, but this Mr. Warren appears to have become a very intimate friend—surprisingly intimate, considering how short a time you have been acquainted with him. He is evidently very often at the corner bungalow; you don't mention the names of any other men who have a similar habit of coming in to see the Doctor, so I presume Mr. Warren is the favoured exception."

There was a good deal more of the same kind, and for a few minutes Kate felt thoroughly indignant and angry with the writer. She had done nothing to justify him in saying such things as these, hinting that she had a warmer regard for Mr. Warren than a girl in her position had any right to entertain. It was unkind and most uncalled for on George's part. She took a step towards her writing-table with the half-formed intention of sitting down there and then to indite such an answer as this letter deserved. But she thought better of it almost as soon as she conceived the idea. Perhaps she had said more than she intended about Mr. Warren, and by careless wording had given a false impression. She had written the letter very late at night when she was sleepy, and no doubt had she kept it back and reread it the following morning she might have detected in it the strain which had roused George's jealousy, and provoked his caustic comments. Yes, she was to blame, not for anything she had done, but for what she had written, and her first aim must be to disabuse her lover's mind of the notion that Mr. Warren was in any way entitled to be considered a peculiarly intimate or favoured friend. And after a little consideration she opened her desk and set to work with this object in view.

"I am afraid my last letter must have given you an absurdly exaggerated idea of our intimacy with this Mr. Warren, particularly of my intimacy with him," she wrote. "It is quite true that he *is* very often here, and that I welcome him when he comes. When I say 'welcome,' I mean that literally and nothing more, for after saying 'how do you do,' and making a remark about the weather, I always leave him and papa to themselves, and come into my own little room next to the drawing-room, where I read, or, more often, write to you. That evening when I last wrote to you papa and Mr. Warren had been sitting in here, because it was wet in the verandah. I joined in the conversation, and had a chat with Mr. Warren afterwards, so I suppose he was for the moment uppermost in my mind. I certainly like him very much, but no better than a score of other men in Meerut. George, how *could* you ever think that this Mr. Warren is more than the most ordinary acquaintance? He *is* almost the only man who comes in with any regularity to see papa, for the simple reason that he is a man papa particularly likes; of course everyone doesn't drop in to see him in the evening so soon after poor Aunt Agnes's death; it's not to be expected that they would. I am ashamed to have written so much about this man, but my last letter seems to have really vexed you, and I cannot bear that you should think I have a single thought for anyone in the world but you."

So Kate Harrison thought she thought, and so she wrote.

Her letter answered its purpose of soothing George Bairdsley's ruffled feelings, for he replied to it by return of post saying that he had done her injustice in writing as he did, and that nothing could shake his faith in her constancy. "The fact is, Kitty," he wrote, "I was feeling a little seedy and out of sorts, and I'm afraid I was in a bad temper and made an ass of myself. I long to get down to you again, but the doctors won't let me go until the cold weather sets in. I've no doubt that when I meet Mr. Warren I shall like him quite as much as you do."

And Kate read the letter with satisfaction and put it away with a pile of others in the same handwriting, telling herself that she need think no more about it. The wrong, if there had been any, was on George's side; he had made the *amende honorable*, and there was nothing more to be said.

Mr. Warren came in that evening, and as the Doctor happened to be engaged at the time he arrived, Kate received him alone.

"What is the news from Simla?" he asked, as he shook hands. "I was sorry to hear from Phelps that Captain Bairdsley was not so well as could be wished a day or two ago."

"Oh, he is all right again, thank you," answered Kate. "He was quite well enough to give me a severe scolding the other day," she added, with a smile.

"Indeed, what have you been doing to deserve that?"

"Well, you will be surprised to hear that it was about you," she replied, wondering, as she spoke, what prompted her to take Mr. Warren into her confidence about the little misunderstanding that had occurred between George and herself.

"About me!" exclaimed Warren. "This kind of thing won't do, Miss Harrison," he added, laughingly.

"Oh, it's all over now," said Kate; "but I was writing to him one evening after we had been having a long talk, and I said too much about you, and made George quite jealous."

Warren laughed again, but not blithely; and it seemed to Kate that he turned to meet her father, who appeared at that moment, with an eagerness which indicated that he wished to avoid hearing or saying more on the subject. She remained in the verandah with the gentlemen, and before long it struck her that Warren was graver and more silent than usual this evening. The Doctor was vigorously denouncing the manner in which the native press of India abused the liberty they enjoyed under British rule, and was eloquent upon some scurrilous diatribes which the *Pioneer* had noticed that day. But Warren, although this same subject had roused his interest to a high pitch only a night or two previously, appeared hardly to follow what his host said, and answered so briefly that his preoccupation soon became apparent to the Doctor too.

"This over-education of the natives will be the ultimate ruin of the country," he was saying. "We are told that public opinion at home cannot be withstood in matters connected with the internal administration of India. But the public at home isn't competent to form an opinion; it can't grasp the thousand and one side-issues and effects of such a movement as this of education. They can't understand at home that we are giving the natives a common ground upon which all races and castes will amalgamate; and, basing their claims upon the knowledge we have put within their reach, assert their demand to be placed in positions for which they are morally unfit. We are undermining caste-prejudices with education, and caste and race-jealousy are our grand safeguards in holding the country."

"I suppose they are," said Warren, speaking as though he had suddenly awakened from sleep to say so.

The Doctor looked a little surprised at the tone and brevity of his answer, and enquired if he had altered or modified the views he had held a few days before.

"On the contrary," replied Warren, "the closer I look into the subject the more strongly I feel upon it." But he spoke as indifferently as if the question was totally void of interest to him.

"The movement is being carried to excess," pursued Dr. Harrison; "we are spending enormous sums annually for the purpose of giving natives ambitions which I sincerely hope we don't mean to gratify; which are utterly impossible to gratify if we mean to maintain our raj."

Warren made some perfunctory reply, and the Doctor attacked the "Education Department" again, holding it solely responsible for the misconduct of native journalists, who used their organs to poison the minds of the enormous mass of the people. He inveighed against it with greater vehemence than he was wont to display upon any subject, but he could not shake his companion out of the constrained silence into which he had fallen.

"The baboo's organ of language is so largely developed that he misleads people who don't know him. I had a beautiful instance of it only yesterday, from one of my clerks who applied for a rise in his pay; he began thus: 'Honoured Sir,—It is a well-known and universally acknowledged fact that the sphere of promotion rotates upon the axis of recommendation.' What do you think of that for a man on thirty rupees a month?"

"That was a flower of rhetoric," said Warren, with a smile.

"It was very happily expressed," answered the Doctor; "but when I came to investigate his claims to promotion, I found that they rested solely on his command of English, which was positively appalling. He used words which I had to hunt up in the dictionary afterwards, for I'd never heard them before. He talked about the inchoation of his career; the value of my arbitrament, and the oblectation with which he should receive it. That's the use a native puts his education to!"

"He is a very wonderful creation, the baboo," said Warren.

"You have some in your office, have you not?" enquired Kate.

"Yes, Miss Harrison; two or three."

He had seemingly tried to avoid speaking to her all the evening, and Kate was at a loss to understand the change in his manner. He was reserved and preoccupied, and answered her almost as briefly as he had done her father. She did her utmost to rouse him, but without success, and finally came to the conclusion that she had somehow given him offence. She could not imagine what she had said to produce this effect upon him, and was disturbed and unhappy, when he got up to go away, bidding a most coldly formal good-night.

"I wonder what has happened to Warren," said her father, as their guest went out of the compound; "I never knew him to be so silent and absent-minded as he was to-night."

"I noticed it, too," said Kate; "he was quite bright and cheery when he came in."

"Something wrong perhaps. No doubt he will be himself again next time he comes in."

Kate hoped so, and retired to her own room wondering whether her remark that George had scolded her upon his account could have had anything to do with his subsequent demeanour. It was very strange: extremely curious. How could it have offended him? She lay awake thinking about it, promising herself that she would take the first opportunity of speaking to him upon the matter. Warren occupied her mind to the total exclusion of all else that night; perhaps that "scolding" was not altogether undeserved.

*(To be continued.)*

